# Difference between continuous positive airway pressure via mask therapy plus chest physiotherapy (CPT) and incentive spirometry therapy plus CPT to treat or prevent acute atelectasis after cardiac surgery

**DOI:** 10.1186/cc10742

**Published:** 2012-03-20

**Authors:** F ALMutairi, S Fallows, W Abukhudair, B Islam

**Affiliations:** 1 University of Chester, Manchester, UK; 2 King Fahd Armed Forces Hospital, Jeddah, Saudi Arabia

## Introduction

All types of therapy such as an incentive spirometry (IS) or continuous positive airway pressure (CPAP) have a valuable role to play in the prevention or the treatment of acute atelectasis. However, the type of therapy that should be used is not yet completely clear. This study aims to clarify the difference in effectiveness between CPAP therapy plus chest physiotherapy (CPT) and IS therapy plus CPT to treat or prevent acute atelectasis.

## Methods

Seventy-two patients who fit the inclusive criteria (smoker, hemodynamically stable, normal lung and above 50 years old) participated in this study. The participants were divided randomly into two groups: the control group used IS 15 times per hour plus CPT 4 hours for 3 days, and the trial group used CPAP via mask therapy for half an hour every 2 hours plus CPT 4 hours. The inspiratory capacity (IC) in liters was used to compare the two groups of therapy and it was measured by incentive spirometer after the operation as baseline test, after 12 hours, 24 hours, 48 hours and post therapy. At the same time, RR, HR and SpO_2 _were measured for both groups. Failure was defined as a need for advanced therapy.

## Results

**Figure 1:**
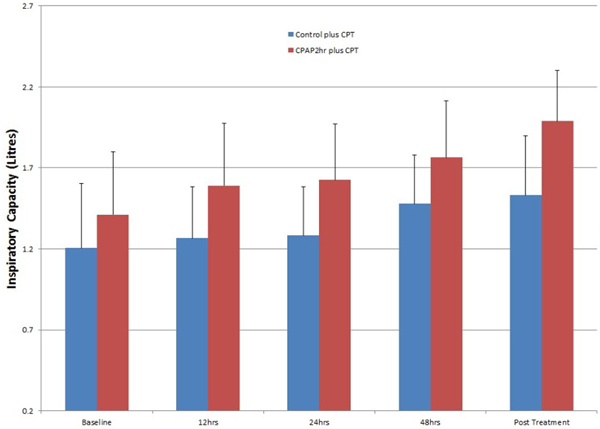
**Effect of adding CPT to CPAP via mask therapy to treat acute atelectasis**.

Thirty-six patients participated in each group (57 male and 15 female). IC was increased significantly in the CPAP group (*P *= 0.005) and SpO_2 _was decreased significantly in the control group (*P *= 0.037). There were no significant differences in RR and HR. See Figure 1.

## Conclusion

Adding chest physiotherapy to CPAP via mask therapy had better outcomes to treat or prevent acute postoperative atelectasis.

